# Burst and Memory-aware Transformer: capturing temporal heterogeneity

**DOI:** 10.3389/fncom.2023.1292842

**Published:** 2023-12-12

**Authors:** Byounghwa Lee, Jung-Hoon Lee, Sungyup Lee, Cheol Ho Kim

**Affiliations:** CybreBrain Research Section, Electronics and Telecommunications Research Institute, Daejeon, Republic of Korea

**Keywords:** burst, temporal heterogeneity, event sequence, timestamp, inter-event time, temporal point process, self-attention, Transformer

## Abstract

Burst patterns, characterized by their temporal heterogeneity, have been observed across a wide range of domains, encompassing event sequences from neuronal firing to various facets of human activities. Recent research on predicting event sequences leveraged a Transformer based on the Hawkes process, incorporating a self-attention mechanism to capture long-term temporal dependencies. To effectively handle bursty temporal patterns, we propose a Burst and Memory-aware Transformer (BMT) model, designed to explicitly address temporal heterogeneity. The BMT model embeds the burstiness and memory coefficient into the self-attention module, enhancing the learning process with insights derived from the bursty patterns. Furthermore, we employed a novel loss function designed to optimize the burstiness and memory coefficient values, as well as their corresponding discretized one-hot vectors, both individually and jointly. Numerical experiments conducted on diverse synthetic and real-world datasets demonstrated the outstanding performance of the BMT model in terms of accurately predicting event times and intensity functions compared to existing models and control groups. In particular, the BMT model exhibits remarkable performance for temporally heterogeneous data, such as those with power-law inter-event time distributions. Our findings suggest that the incorporation of burst-related parameters assists the Transformer in comprehending heterogeneous event sequences, leading to an enhanced predictive performance.

## 1 Introduction

Temporal heterogeneity is frequently referred to as *burst* within the context of complex systems. Numerous natural and social phenomena exhibit bursty temporal patterns such as single-neuron firing (Kemuriyama et al., 2010; Chan et al., 2016; Metzen et al., 2016; Zeldenrust et al., 2018), earthquakes (Corral, 2004; de Arcangelis et al., 2006), solar flares (Wheatland et al., 1998), and human activity (Barabasi, 2005; Karsai et al., 2018). The term temporal heterogeneity rigorously implies that the distribution of inter-event times, which is the time intervals between two consecutive events, exhibits a heavy-tailed distribution such as a power-law distribution. Moreover, when the system is generally temporally heterogeneous, it implies the presence of temporal correlations among inter-event times. For example, the inter-spike interval distribution display temporally heterogeneous patterns, which cannot be simply interpreted as a random or regular process. Numerous studies have addressed temporal correlations between bursty spikes using approaches such as the non-renewal process (Shahi et al., 2016), intensity functions with voltage-dependent terms (Yamauchi et al., 2011), and transitions between burst and non-burst states (Dashevskiy and Cymbalyuk, 2018). To quantify temporal heterogeneity, two commonly employed single-value metrics are burstiness and memory coefficient.

**Figure 1:**
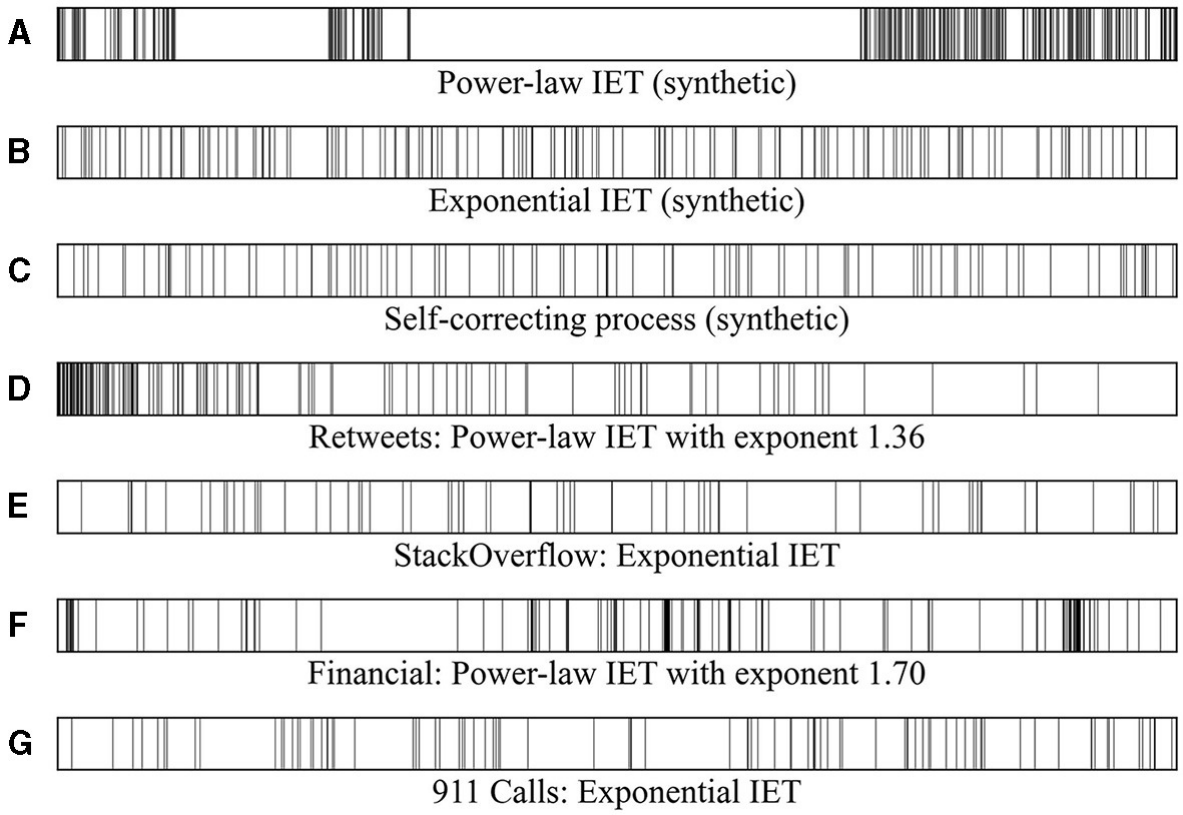
**(A, D, F)** Heterogeneous event sequences with a power-law inter-event time distribution. These event sequences exhibit a high burstiness parameter with significant temporal heterogeneity. **(B, C, E, G)** Event sequences with an exponential inter-event time distribution. These event sequences have burstiness parameters close to 0 and memory coefficients clustered around 0.

Figure 1 illustrates the distinction between temporally heterogeneous inter-event times and those that tend toward homogeneity. The event sequences in Figures 1A, D, F serve as examples of temporal heterogeneity with a power-law inter-event time distribution. The event sequences in Figures 1B, C, E, G present instances that exhibit a more homogeneous random characteristic with an exponential inter-event time distribution. Evidently, the bursty event sequence exhibits clustered events within burst trains, in contrast to the non-burst sequence. Such uneven event occurrences can affect the prediction of event sequences. Without properly accounting for the complicated correlation structure and heterogeneity therein, naive models may struggle to effectively discern hidden patterns.

Event sequence data encompass the temporal occurrences of events spanning various domains, ranging from natural phenomena to social activities. Unlike time series data, event sequence data are defined by sequentially ordered timestamps that signify the timing of individual event occurrences. Numerous studies have focused on predicting the timing of subsequent events have been conducted using temporal point processes (TPPs) (Daley and Vere-Jones, 2008). One of the most widely employed TPP is the Hawkes process (Hawkes, 1971). This process embodies a self-exciting mechanism, wherein preceding events stimulate the occurrence of subsequent events. In contrast to the Hawkes process, the self-correcting process provides a feasible method for establishing regular point patterns (Isham and Westcott, 1979).

The Poisson point process can be employed to generate entirely random and memory-less events (Kingman, 1992). In the Poisson process, the inter-event time (IET) follows an exponential distribution. The Cox process is a generalized Poisson process in which the intensity function varies with the stochastic process (Cox, 1955); thus, it is also referred to as a doubly stochastic Poisson process. Cox processes are frequently employed to model and predict the arrival of insurance claims, enabling insurers to assess risk and manage resources effectively (Rolski et al., 2009). If the intensity function is not entirely random, as in the Cox process, but given as a deterministic time-varying function, it is referred to as an inhomogeneous Poisson process.

Leveraging advancements in deep neural networks, recent studies have introduced Hawkes process models based on neural network frameworks. Specifically, the models of Marked Temporal Point Processes (RMTPP) (Du et al., 2016) and Continuous Time LSTM (CTLSTM) (Mei and Eisner, 2017), utilizing Recurrent Neural Networks (RNN) and Long Short-Term Memory (LSTM) (Hochreiter and Schmidhuber, 1997), exhibited better performance than Hawkes processes. More recently, the Transformer Hawkes Process (THP) (Zuo et al., 2020) and the Self-Attentive Hawkes Process (SAHP) (Zhang et al., 2020), both grounded in self-attention mechanisms, have demonstrated improved performance.

**Figure 2:**
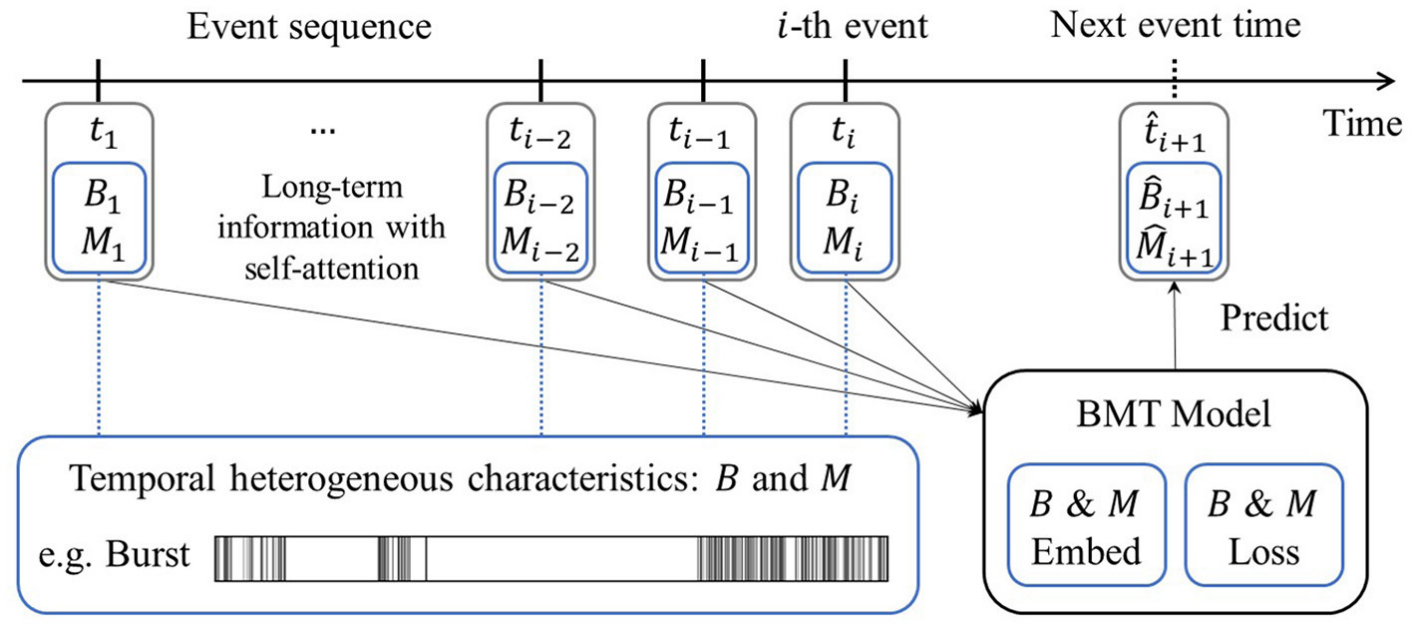
Schematic diagram of the Burst and Memory-aware Transformer model. Leveraging information from the preceding events, including burstiness *B* and memory coefficient *M*, the model predicts the timing of the next event through *B* & *M* embedding and the corresponding *B* & *M* loss.

Our research was primarily motivated by the idea that incorporating temporal heterogeneous characteristics into event sequence predictions yields a superior performance in forecasting events. We propose a *Burst and Memory-aware Transformer* (BMT) model, signifying its capability to train the Transformer by leveraging insights derived from burstiness and memory coefficient, both of which are associated with temporal heterogeneity. Notably, these two metrics were incorporated as embedding inputs for the Transformer architecture. Moreover, a loss function related to these metrics was formulated and employed, thereby enabling the model to naturally capture temporal heterogeneity. The overall schematic diagram of the BMT model is depicted in Figure 2.

The main contributions of this paper is summarized as follows:

- The BMT model was developed to integrate insights from the complex systems theory into the Transformer-based temporal point process model, enhancing the capability to incorporate temporal heterogeneity. This study offers a preliminary approach to connect these two distinct disciplines.
- The BMT model surpasses state-of-the-art models by effectively integrating burstiness and memory coefficient into both the embedding procedure and associated loss functions. This is confirmed through extensive numerical experiments across a range of scenarios, including those with and without burstiness and memory coefficient embedding and related loss functions, using real-world datasets and synthetic datasets generated via a copula-based algorithm.
- Our investigation revealed that the BMT model offers particular advantages when dealing with temporally heterogeneous data, such as datasets characterized by a power-law inter-event time distribution, commonly observed in bursty event sequences.
- Our research indicates that excluding either burstiness and memory coefficient embedding or their corresponding loss functions leads to a noticeable reduction in performance. This emphasizes the imperative nature of integrating both elements to achieve optimal performance.

In cases where the inter-event time distribution of the target event sequence exhibits a heavy-tailed distribution, such as a power-law distribution, or where the values of burstiness and memory coefficient significantly deviate from zero, the BMT model ensures superior performance compared to basic Transformer-based models.

The structure of the paper is outlined as follows: Section 2 introduces the background pertaining to the temporal point process, temporal heterogeneity, and generating method for synthetic datasets; Section 3 introduces our Burst and Memory-aware Transformer model; Section 4 presents numerical experiments on synthetic and real-world datasets; Section 5 presents the performance evaluation results; and Section 6 presents the conclusion.

## 2 Background

### 2.1 Temporal point process

A temporal point process (TPP) is a stochastic process involving the occurrence of multiple events as time progresses. The foundational data employed to construct the TPP model consists of event sequence data, encompassing event times ${\left\{t_{i}\right\}}_{i=1}^{n}$ along with optional marks ${\left\{\kappa_{i}\right\}}_{i=1}^{n}$. For example, spike train sequences of neurons are composed of timings of occurrences, along with action potential as associated marks.

In this study, we examine the unmarked case to specifically investigate the effects of burst and memory phenomena, while excluding the influence of correlations with marks that do not align with the research direction. For the prediction of the marked TPP model, one approach involves the independent modeling of the target's marks by thresholding. Alternatively, based on contextual analysis (Jo et al., 2013), interactions with multiple neighbors within an egocentric network can be considered as marks and subsequently modeled.

TPP encompasses the modeling of the conditional intensity function $\lambda \left(t|H_{t}\right)$ given the history of event times $H_{t}\equiv \left(t_{1},...t_{n}\right)$. The notation for the history of event times, $H_{t}$ will be omitted for convenience. The intensity function characterizes the instantaneous event rate at any given time by considering past event occurrences. The probability density function *P*(*t*) and cumulative distribution function *F*(*t*) can be derived based on the intensity function, as follows (Rasmussen, 2018):


(1) $$\begin{array}{l} P\left(t\right)=\lambda \left(t\right)\exp\left(-\int_{t_{i-1}}^{t}\lambda \left(t^{\prime }\right)dt^{\prime }\right), \end{array}$$

(2) $$\begin{array}{l} F\left(t\right)=1-\exp\left(-\int_{t_{i-1}}^{t}\lambda \left(t^{\prime }\right)dt^{\prime }\right). \end{array}$$

#### 2.1.1 Hawkes process

The Hawkes process, also known as the self-exciting point process, is for a situation where a preceding event excites the occurrence of a subsequent event (Hawkes, 1971). The intensity function λ(*t*) of the Hawkes process is defined as


(3) $$\begin{array}{l} \lambda \left(t\right)=\zeta +\eta \underset{t_{i}<t}{\sum }\exp\left(-\left(t-t_{i}\right)\right), \end{array}$$

where the base intensity ζ and η are positive parameters. When a new event occurs during this process, the intensity increases with η and immediately decays exponentially. The probability of the next event occurring is highest immediately following the incidence of the previous event, and it gradually decreases as time elapses. As a result, this process causes events to cluster together. This includes events that happen quickly in a short time and then long times when nothing happens. The generalized Hawkes process is defined as follows:


(4) $$\begin{array}{l} \lambda \left(t\right)=\zeta +\eta \underset{t_{i}<t}{\sum }\gamma \left(t-t_{i}\right), \end{array}$$

where ζ ≥ 0, η > 0, and γ(*t*) is a density on (0, ∞).

#### 2.1.2 Self-correcting process

In contrast to the Hawkes process, the self-correcting process generates regular inter-event time sequences with randomness (Isham and Westcott, 1979). The intensity function λ(*t*) for the self-correcting process is defined as follows:


(5) $$\begin{array}{l} \lambda \left(t\right)=\exp\left(\zeta t-\underset{t_{i}<t}{\sum }\eta \right), \end{array}$$

where ζ and η are positive parameters.

#### 2.1.3 Neural Hawkes process

A limitation of the Hawkes process is that the preceding event cannot inhibit the occurrence of a subsequent event. To overcome these limitations, the neural Hawkes process, which considers the nonlinear relationship with past events using recurrent neural networks, was introduced (Mei and Eisner, 2017). The intensity function λ(*t*) for the neural Hawkes process is defined as follows:


(6) $$\begin{array}{l} \lambda \left(t\right)=f({\text{w}}^{⊤}\text{h}\left(t\right)), \end{array}$$

where *f*(*x*) = βlog(1 + exp(*x*/β)) is the softplus function with parameter β which guarantees a positive intensity, and **h**(*t*)s are hidden representations of the event sequence from a continuous-time LSTM model. Here, the intensity we refer to is not the marked intensity λ_*k*_; Instead, our focus is on the inherent temporal heterogeneity structure, excluding any interference from correlations between event types and times.

#### 2.1.4 Transformer-based Hawkes process

The Transformer is a deep learning architecture for sequence processing such as natural language processing, with a multi-head self-attention module that captures long-range dependencies within sequences (Vaswani et al., 2017). The Transformer is used not only in language models but also in computer vision, audio processing, and time series forecasting (Lim et al., 2021; Wen et al., 2022; Ma et al., 2023). Recently, the Transformer architecture has also been applied to modeling temporal point processes. The Transformer Hawkes Process (Zuo et al., 2020) and the Self-Attentive Hawkes Process (Zhang et al., 2020) were introduced to model the Hawkes process with a self-attention mechanism to capture the long-range correlations underlying both event times and types.

THP and SAHP differ in two aspects: their use of positional encoding and the form of the intensity function. SAHP employs time-shifted positional encoding to address the limitations of conventional methods, which solely account for the sequence order and neglect inter-event times. The intensity function of the THP model is the softplus function of the weighted sum of three terms: ratio of elapsed time from the previous event, hidden representation vector from the encoder, and base. Conversely, the intensity function of the SAHP model is formulated as a softplus of the Hawkes process terms, each of which is derived from the scalar transformation and nonlinear activation function applied to the hidden representation vector from the encoder.

For both the THP and SAHP models, across synthetic and real-world datasets, their performances in event type prediction and event time prediction surpassed that of the baseline model: Hawkes Process as described in Equation (3), Fully Neural Network model (Omi et al., 2019), Log-normal Mixture model (Shchur et al., 2019), Time Series Event Sequence (TSES) (Xiao et al., 2017), Recurrent Marked Temporal Point Processes (Du et al., 2016), and Continuous Time LSTM (Mei and Eisner, 2017). Given the superior performance of THP over the remaining baseline models, this study refrains from direct performance comparison with the SAHP and baseline models (Zuo et al., 2020), opting to concentrate exclusively on performance comparison with the THP model.

### 2.2 Temporal heterogeneity

Temporal heterogeneity or burst is characterized by various metrics. The most fundamental quantity is the probability density function of the inter-event times. The inter-event time is defined as the time interval between two consecutive events, that is, τ_*i*_ ≡ *t*_*i*+1_ − *t*_*i*_, where *t*_*i*_ is *i*-th event time of the event sequence.

When the inter-event time distribution is heavy-tailed, the corresponding event sequence exhibits temporal heterogeneity. Specifically, the power-law inter-event distribution found in diverse natural and social phenomena is as follows:


(7) $$\begin{array}{l} P\left(\tau \right)\sim \tau^{-\alpha }, \end{array}$$

where *a* is a constant and α is a power-law exponent.

#### 2.2.1 Burstiness parameter

Several metrics characterize the properties of temporal heterogeneity. Burstiness *B* measures the burst phenomenon (Goh and Barabási, 2008), and is defined as follows:


(8) $$\begin{array}{l} B\equiv \frac{r-1}{r+1}=\frac{\sigma -\langle \tau \rangle }{\sigma +\langle \tau \rangle }, \end{array}$$

where *r* ≡ σ/〈τ〉 is the coefficient of variation (CV) of the inter-event time and σ and 〈τ〉 is the standard deviation and average of τs, respectively. Here, *B* = −1 for regular event sequences, *B* = 0 for Poissonian random cases, and *B* = 1 for extremely bursty cases.

When the number of events is sufficiently small, the burstiness parameter causes errors. The fixed burstiness parameter considering the finite-size effect is as follows (Kim and Jo, 2016):


(9) $$\begin{array}{l} B_{n}\equiv \frac{\sqrt{n+1}r-\sqrt{n-1}}{\left(\sqrt{n+1}-2\right)r+\sqrt{n-1}}. \end{array}$$

We employed the fixed burstiness parameter (9) to handle short-length event sequences throughout this study.

#### 2.2.2 Memory coefficient

The memory coefficient *M* quantifies the correlations between consecutive inter-event times within a sequence consisting of *n* inter-event times, that is, {_τ_*i*_}*i* = 1, ..., *n*_, as follows (Goh and Barabási, 2008):


(10) $$\begin{array}{l} M\equiv \frac{1}{n-1}\overset{n-1}{\underset{i=1}{\sum }}\frac{\left(\tau_{i}-{\langle \tau \rangle }_{1}\right)\left(\tau_{i+1}-{\langle \tau \rangle }_{2}\right)}{\sigma_{1}\sigma_{2}}, \end{array}$$

where 〈τ〉_1_ (〈τ〉_2_) and σ_1_ (σ_2_) are the average and standard deviation of the inter-event times τ_1_, τ_2_, ..., τ_*n*−1_ (τ_2_, τ_3_, ..., τ_*n*_), respectively. This is the Pearson correlation coefficient between consecutive inter-event times. Here, *M* = 0 indicates no correlation, and *M* > 0 indicates a positive correlation, which means that a large inter-event time follows after a large inter-event time and vice versa for small inter-event time. *M* < 0 indicates a negative correlation, which means small inter-event time follows after the large inter-event time and vice versa for a large inter-event time.

#### 2.2.3 Applications of *B* and *M* to BMT model

When plotting *M* on the *x*-axis and *B* on the *y*-axis for datasets with various inter-event time distributions, it can be observed that event sequences with similar inter-event time distributions tend to cluster at similar positions (Goh and Barabási, 2008). Essentially, if the ranges of *B* and *M* values are known, a rough estimate of the inter-event time distribution can be anticipated. Building on this insight, we devised a BMT model to facilitate learning by designing a method in which the values of *B* and *M* were combined and fed into the encoder as inputs. Specifically, when the values of *B* and *M* exhibit temporal heterogeneity in their ranges, the encoder of the Transformer can produce inter-event time prediction values with a heavy-tailed inter-event time distribution.

Moreover, *B* and *M* are not independent: they are intertwined and move in conjunction. For instance, even when attempting to alter only *M* by shuffling the inter-event times, *B* can also change. This serves as evidence that embedding both *B* and *M* concurrently yields superior performance compared with embedding either one of them individually.

### 2.3 Copula-based algorithm for generating sequence of inter-event times

To comprehend the impact of burstiness and memory coefficient on the model, we generated synthetic datasets using a copula-based algorithm (Jo et al., 2019). The content of the copula-based algorithm in this study was obtained from Jo et al. (2019). For convenience, we provide a brief overview of the relevant content. The copula-based algorithm models the joint probability distribution of two consecutive inter-event times, that is, *P*(τ_*i*_, τ_*i*+1_), by adopting the Farlie-Gumbel-Morgenstern (FGM) copula (Nelsen, 2006). The joint probability distribution according to the FGM copula is formulated as follows:


(11) $$\begin{array}{l} P\left(\tau_{i},\tau_{i+1}\right)=P\left(\tau_{i}\right)P\left(\tau_{i+1}\right)\left[1+rf\left(\tau_{i}\right)f\left(\tau_{i+1}\right)\right], \end{array}$$

where


(12) $$\begin{array}{l} f\left(\tau \right)\equiv 2F\left(\tau \right)-1,\;F\left(\tau \right)\equiv \int_{0}^{\tau }d\tau^{\prime }P\left(\tau^{\prime }\right). \end{array}$$

Parameter *r* is used to control the correlation between τ_*i*_ and τ_*i*+1_ and is in the range of −1 ≤ *r* ≤ 1. *F*(τ) is the cumulative distribution function (CDF) of *P*(τ). After applying the transformation method (Clauset and Shalizi, 2009), the next inter-event time τ_*i*+1_ can be obtained as Jo et al. (2019)


(13) $$\begin{array}{l} \tau_{i+1}=F^{-1}\left[\frac{c_{i}-1+\sqrt{{\left(c_{i}+1\right)}^{2}-4c_{i}x}}{2c_{i}}\right], \end{array}$$

where *F*^−1^ is the inverse of *F*(τ), *c*_*i*_ ≡ *rf*(τ_*i*_), and *x* is a random number sampled from a uniform distribution within interval 0 ≤ *x* < 1. The copula-based algorithm has the advantage of generating event sequences with independent control of the inter-event time distribution and memory coefficient.

## 3 Burst and Memory-aware Transformer

### 3.1 Discretization of *B* and *M*

Given that the burstiness parameter and memory coefficient are real numbers within the range of [−1, 1], it is necessary to discretize them for embedding within the Transformer. We adopt the uniform discretization transform; the range [−1, 1] is divided into segments of fixed length by the number of bins *b*, respectively, and subsequently mapped to a single natural number. The continuous values of the burstiness parameter *B* and memory coefficient *M* are discretized into natural numbers *d*_*B*_ and *d*_*M*_, respectively. For example, when the number of bins is *b* = 4, then *d*_*B*_ = 3 if *M* = 0.2, and it *d*_*B*_ = 1 if *M* = −0.7. Then, one can obtain the discretized pairs of *B* and *M* as (*d*_*B*_, *d*_*M*_), where *d*_*B*_ and *d*_*M*_ are ranging from 1 to *b*. To map the pair into a unique natural number, the Cantor pairing function was employed. The Cantor pairing function maps discretized *d*_*B*_ and *d*_*M*_ into a unique natural number *d*_*B,M*_ as


(14) $$\begin{array}{l} d_{B,M}\equiv \frac{1}{2}\left(d_{B}+d_{M}\right)\left(d_{B}+d_{M}+1\right)+d_{M}. \end{array}$$

When the number of discretization bins is *b*, the number of *d*_*B,M*_ is *b*^2^, corresponding to the vocabulary size of the Transformer. Then, we can obtain the one-hot vector of the discretized *B* & *M* as ${\text{d}}_{B,M}\in {\mathbb{R}}^{b^{2}}$.

### 3.2 Embedding event times, and *B* and *M*

The event sequence $S={\left\{t_{i}\right\}}_{i=1}^{n}$ of *n* events and discretized and one-hot *B* & *M*, **d**_*B,M*_ are fed into the self-attention module after proper encoding. First, the event times are transformed using the positional encoding method (Vaswani et al., 2017) to embed the temporal order information into an event sequence. The *j*-th element of sinusoidal positional encoding for the *i*-th event time *t*_*i*_ is calculated as:


(15) $$\begin{array}{l} {\left[{\text{z}}_{t}\left(t_{i}\right)\right]}_{j}=\{\begin{array}{c} \sin\left(\omega_{k}t_{i}\right),\;\text{if }j=2k\\ \cos\left(\omega_{k}t_{i}\right),\;\text{if }j=2k+1, \end{array} \end{array}$$

where $\omega_{k}=1/10,000^{2k/d}$, the embedding index *k* is the quotient when dividing *j* by 2, and ${\text{z}}_{t}\left(t_{i}\right)\in {\mathbb{R}}^{d}$, where *d* is the encoding dimension. By multiplying ω_*k*_ with the event time *t*_*i*_, it is converted into an angle, which is then mapped to sine and cosine functions, providing different positional information for each event time.

For the given event times ${\left\{t_{i}\right\}}_{i=1}^{n}$, the inter-event times are τ_*i*_ ≡ *t*_*i*+1_ − *t*_*i*_ for *i* = 1, ..., *n* − 1. The burstiness parameter (9) and memory coefficient (10) were calculated for all partial sequences. This essentially implies that the input to the encoder is fed sequentially from *t*_1_, ..., *t*_*i*_,..., *t*_*n*_, and for each of these instances, the *B* & *M* embedding incorporates the calculated *B* and *M* values up to *t*_1_ (i.e., *B*_1_ and *M*_1_), ..., up to *t*_*i*_ (i.e., *B*_*i*_ and *M*_*i*_), ..., and up to *t*_*n*_ (*B*_*n*_ = *B* and *M*_*n*_ = *M* for the entire sequence). Note that, during the actual operation of the Transformer, computations are performed in parallel; thus, the sliding *B* & *M* embedding vectors form a lower triangular matrix.

The *B* & *M* embedding vector **z**_*e*_(*B*_*i*_, *M*_*i*_) for the one-hot vector of the discretized *B*_*i*_ and *M*_*i*_, **d**_*B*_*i*_, *M*_*i*__, is calculated using a linear embedding layer as follows:


(16) $$\begin{array}{l} {\text{z}}_{e}\left(B_{i},M_{i}\right)={\text{W}}_{E}{\text{d}}_{B_{i},M_{i}}, \end{array}$$

where ${\text{W}}_{E}\in {\mathbb{R}}^{d\times b^{2}}$ denotes an embedding matrix. Then for the *i*-th event, the event time embedding vector ${\text{z}}_{t}\left(t_{i}\right)\in {\mathbb{R}}^{d}$ and the *B* & *M* embedding vector ${\text{z}}_{e}\left(B_{i},M_{i}\right)\in {\mathbb{R}}^{d}$ are summed together to acquire the hidden representation of the *i*-th event ${\text{z}}_{i}\in {\mathbb{R}}^{d}$ as:


(17) $$\begin{array}{l} {\text{z}}_{i}={\text{z}}_{t}\left(t_{i}\right)+{\text{z}}_{e}\left(B_{i},M_{i}\right). \end{array}$$

Then the embedding matrix for a whole single event sequence is given by:


(18) $$\begin{array}{l} \text{Z}={\left[{\text{z}}_{i}\right]}_{i=1,...,n}, \end{array}$$

where **Z** ∈ ℝ^*n*×*d*^ and *n* is the length of the event sequence, that is, the number of events in a single sequence.

### 3.3 Self-attention module

After acquiring the embedding matrix **Z** for each event sequence according to Equation (18), we propagated **Z** into the input of the self-attention module. The resulting attention output **S** is defined as follows:


(19) $$\begin{array}{l} \text{S}=\text{Softmax}\left(\frac{\text{Q}{\text{K}}^{⊤}}{\sqrt{d_{K}}}\right)\text{V}, \end{array}$$

where **Q** = **ZW^Q^**, **K** = **ZW^K^**, **V** = **ZW^V^**, and $\text{S}\in {\mathbb{R}}^{n\times d_{V}}$. Here, **Q**, **K**, and **V** represent the query, key, and value matrices, respectively, obtained by applying distinct transformations to **Z**. The transformation parameters are ${\text{W}}^{Q}\in {\mathbb{R}}^{d\times d_{K}},{\text{W}}^{K}\in {\mathbb{R}}^{d\times d_{K}}$, and ${\text{W}}^{V}\in {\mathbb{R}}^{d\times d_{V}}$, respectively. In contrast to conventional RNN models, the self-attention mechanism enables an equitable comparison of not only recent values but also the significance of distant past values of the sequence. Consequently, this facilitates the learning of long-term dependencies.

The BMT model employs multi-head attention, similar to other Transformers. Multi-head attention enables the model to manage diverse patterns and contexts of the input sequence. The multi-head attention output **S** is given by **S** = [**S**_1_, ..., **S**_*i*_, ..., **S**_*m*_]**W**_*O*_, where ${\text{S}}_{i}\in {\mathbb{R}}^{n\times d_{V}/m}$ is the attention output for the *i*-th multi-head and ${\text{W}}_{O}\in {\mathbb{R}}^{m\cdot d_{V}\times d}$ is aggregation parameters.

After the multi-head attention, the resulting attention output **S** is subsequently passed into a position-wise feed-forward network, yielding hidden representations **h**(*t*) for the event sequence as:


(20) $$\begin{array}{l} \text{H}=\text{ReLU}\left(\text{S}{\text{W}}_{\text{FC1}}+{\text{b}}_{1}\right){\text{W}}_{\text{FC2}}+{\text{b}}_{2}, \end{array}$$

where ${\text{W}}_{\text{FC1}}\in {\mathbb{R}}^{d\times d_{H}},{\text{W}}_{\text{FC2}}\in {\mathbb{R}}^{d_{H}\times d},{\text{b}}_{1}\in {\mathbb{R}}^{d_{H}}$, and ${\text{b}}_{2}\in {\mathbb{R}}^{d}$ are the parameters of each neural network. The *i*-th event of the event sequence corresponds to the *i*-th row of the hidden representation matrix **H**, that is, **h**(*t*_*i*_) = **H**(*i*, :). Furthermore, masks are employed to prevent the model from learning about the future in advance. The hidden representation **H** ∈ ℝ^*n*×*d*^ encapsulates insights regarding burstiness and memory coefficient for each event within the sequence, acquired through the self-attention mechanism. We further enhanced the incorporation of sequential information by applying LSTM to the hidden representation.

### 3.4 Training and loss function

The BMT model employs five types of loss functions: (1) squared error of the event time, (2) event log-likelihood loss as described in Equation (22), (3) cross entropy of discretized *B* & *M*, (4) squared error of *B*, and (5) squared error of *M*.

#### 3.4.1 Event time loss

The most crucial loss function within the model is how accurately it predicts the next event times. The next event time prediction is ${\hat{t}}_{i+1}={\text{W}}_{t}\text{h}\left(t_{i}\right)$, where ${\text{W}}_{t}\in {\mathbb{R}}^{1\times d}$ is the parameter of the event time predictor. To address this, the squared error loss function of the event times for the event sequence is defined as:


(21) $$\begin{array}{l} L_{t}=\overset{n}{\underset{i=2}{\sum }}{\left(t_{i}-{\hat{t}}_{i}\right)}^{2}, \end{array}$$

where ${\hat{t}}_{i}$ is the predicted event time.

#### 3.4.2 Event log-likelihood

The typical approach for optimizing the parameters of the Hawkes process involves utilizing Maximum Likelihood Estimation (MLE). There are two constraints: (1) no events before time 0, and (2) unobserved event time must appear after the observed time interval. When the observed event sequences are *t*_1_, ..., *t*_*n*_ ∈ [0, *T*), then likelihood of an event sequence is given by ${ℒ}^{\prime }=P(t_{1})\cdots P(t_{n-1})(1-F(T)),\text{ where }F(\cdot )$ is the cumulative distribution function, and the last term is for the second constraint. Using (1) and (2), and applying the logarithm function, we obtain the following log-likelihood:


(22) $$\begin{array}{l} {ℒ}_{\lambda }=\sum_{i=1}^{n}\log\lambda (t_{i})-\int_{0}^{T}\lambda (t^{\prime })dt^{\prime }. \end{array}$$

The first term denotes the sum of the log-intensity functions for the past *n* events, and the second term represents the non-event log-likelihood.

Here, the intensity function λ(*t*) is defined in the interval *t* ∈ [*t*_*i*_, *t*_*i*+1_] according to the following expression:


(23) $$\begin{array}{l} \lambda \left(t\right)=\beta \log\left(1+\exp\left({\text{w}}_{\lambda }^{⊤}\text{h}\left(t_{i}\right)\beta^{-1}\right)\right), \end{array}$$

where β is the softness parameter, ${\text{w}}_{\lambda }\in {\mathbb{R}}^{d\times 1}$ is a parameter that converts the term inside the exponential function into a scalar, and **h** is the hidden representation derived from the encoder. The essence of this intensity function aligns with that of the Neural Hawkes Process, as shown in Equation (6). The softplus function formulation was employed to guarantee non-negativity of the intensity.

#### 3.4.3 Discretized *B* and *M* loss

The model predicts the discretized ${\hat{B}}_{i}$ & ${\hat{M}}_{i}$, ${\hat{d}}_{B_{i},M_{i}}$, based on the hidden representations **h**(*t*_*i*−1_) as:


(24) $$\begin{array}{l} {\hat{\text{p}}}_{i}=\text{Softmax}\left({\text{W}}_{B,M}\text{h}\left(t_{i-1}\right)\right), \end{array}$$

(25) $$\begin{array}{l} {\hat{d}}_{B_{i},M_{i}}=\arg\underset{d^{\prime }}{\max}{\hat{\text{p}}}_{i}\left(d^{\prime }\right), \end{array}$$

where ${\text{W}}_{B,M}\in {\mathbb{R}}^{b^{2}\times d}$ is the parameter of the discretized *B*_*i*_ & *M*_*i*_ predictor, and ${\hat{\text{p}}}_{i}\left(d^{\prime }\right)$ is the *d*′-th element of ${\hat{\text{p}}}_{i}$. To measure the accuracy of *B*_*i*_ & *M*_*i*_ embedding, the following cross-entropy between the ground truth discretized *B*_*i*_ & *M*_*i*_, *d*_*B*_*i*_, *M*_*i*__, and the predicted discretized ${\hat{B}}_{i}$ & ${\hat{M}}_{i}$, ${\hat{d}}_{B_{i},M_{i}}$, is calculated:


(26) $$\begin{array}{l} L_{B,M}=-\overset{n}{\underset{i=2}{\sum }}{\text{d}}_{B_{i},M_{i}}^{⊤}\log\left({\hat{\text{p}}}_{i}\right), \end{array}$$

where ${\text{d}}_{B_{i},M_{i}}\in {\mathbb{R}}^{b^{2}}$ is the ground truth one-hot encoding vector.

#### 3.4.4 *B* loss and *M* loss

Additionally, the model utilizes the squared errors of the burstiness parameter directly as:


(27) $$\begin{array}{l} L_{B}=\overset{n}{\underset{i=2}{\sum }}{\left(B_{i}-{\hat{B}}_{i}\right)}^{2}, \end{array}$$

where *B*_*i*_ and ${\hat{B}}_{i}$ are the ground truth and predicted burstiness parameters, respectively. The squared errors of the memory coefficient value can be defined in a similar manner.


(28) $$\begin{array}{l} L_{M}=\overset{n}{\underset{i=2}{\sum }}{\left(M_{i}-{\hat{M}}_{i}\right)}^{2}, \end{array}$$

where *M*_*i*_ and ${\hat{M}}_{i}$ is ground truth and predicted memory coefficient, respectively.

#### 3.4.5 Overall loss

**Figure 3:**
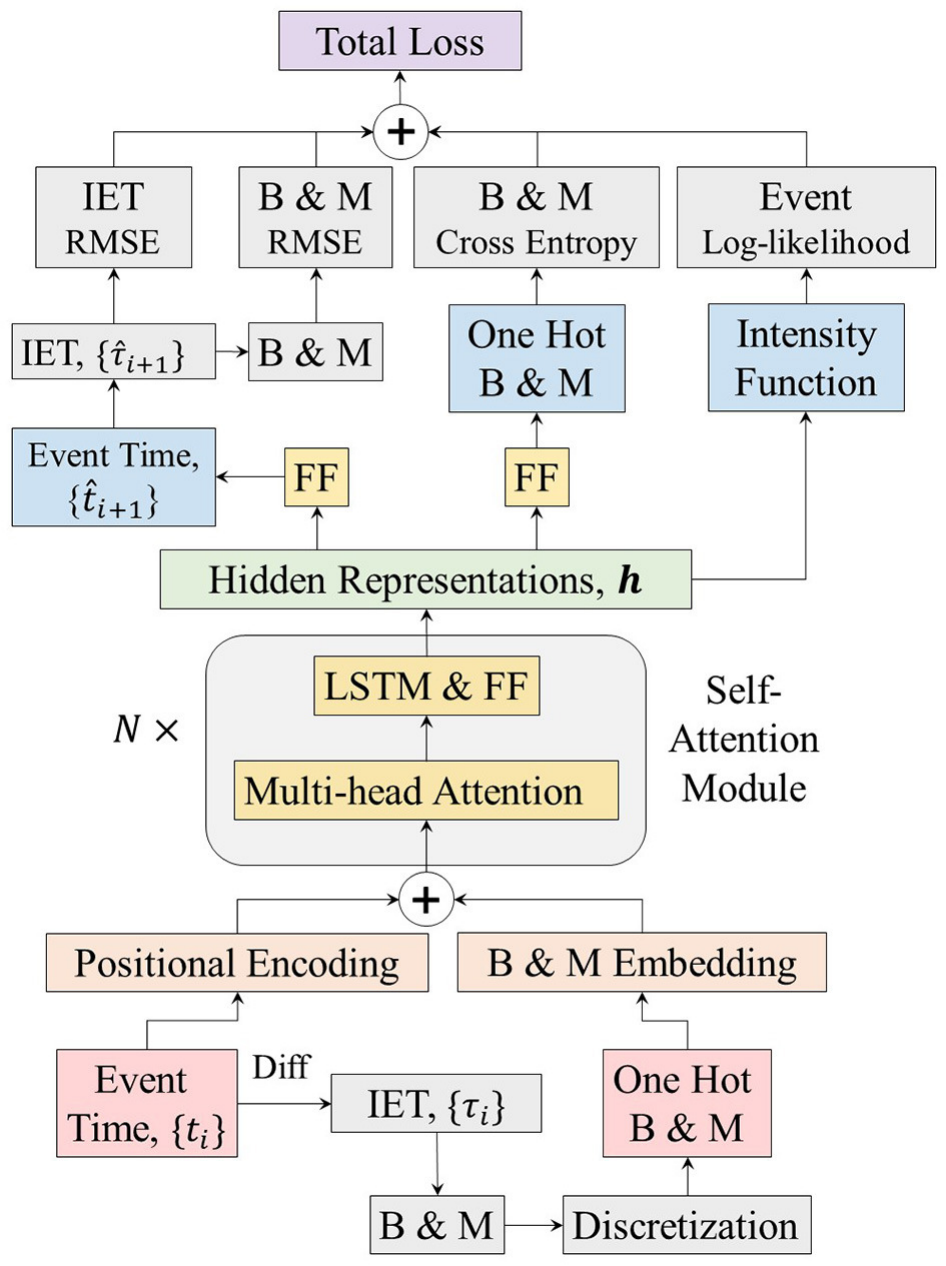
Architecture of the Burst and Memory-aware Transformer model. IET; inter-event time; FF, feed-forward neural network; *B*, burstiness; *M*, memory coefficient.

By aggregating the aforementioned loss functions (21), (22), and (26)–(28), the overall loss function of the model is defined as follows:


(29) $$\begin{array}{l} L=L_{t}+\alpha_{1}L_{\lambda }+\alpha_{2}L_{B,M}+\alpha_{3}L_{B}+\alpha_{4}L_{M}, \end{array}$$

where α_1_ to α_4_ are the hyperparameters that balance each loss function determined using the validation datasets. The overall framework of the BMT model is illustrated in Figure 3.

## 4 Experiments

### 4.1 Synthetic datasets

We generated synthetic data using the copula-based algorithm for two different inter-event time distributions. The model was tested for the exponential and power-law inter-event time distribution, which also have a different range of memory coefficient and burstiness, to directly understand the impact of temporal heterogeneity on the BMT model and other models. Along with the two synthetic datasets below, we tested the regular event sequences generated by the self-correcting process, as in Equation (5). The statistics of the datasets are displayed in Table 1.

**Table 1:** Datasets statistics.

**Datasets**		**Power-law**	**Exponential**	**Self-correcting**	**Retweets**	**Stack Overflow**	**Financial**	**911 Calls**
IET	Mean	3.4645	57.495	0.20015	2,840.8	0.58677	1.5853	338.14
	S.D.	2.9372	32.938	0.00193	2,157.8	0.16667	5.6152	249.20
B	Mean	0.176	−0.008	−0.048	0.754	0.052	0.522	0.100
	S.D.	0.335	0.068	0.028	0.137	0.083	0.069	0.100
M	Mean	−0.021	−0.059	−0.084	0.442	0.031	0.155	0.009
	S.D.	0.195	0.200	0.058	0.274	0.119	0.078	0.158

IET, inter-event time; B, burstiness; M, memory coefficient; S.D., standard deviation.

#### 4.1.1 Power-law inter-event time distribution

The power-law inter-event time distribution with a power-law exponent α is defined as *P*(τ) = (α − 1)τ^−α^θ(τ − 1) and the corresponding cumulative distribution function is *F*(τ) = (1 − τ^1−α^)θ(τ − 1), where θ(·) represents the Heaviside step function with a lower bound of 1. After substituting the inter-event time distribution into Equation (13), we obtain the next inter-event time τ_*i*+1_ from a given previous inter-event time τ_*i*_ and random number *x* in 0 ≤ *x* < 1 as


(30) $$\begin{array}{l} \tau_{i+1}={\left[\frac{2c_{i}}{c_{i}+1-\sqrt{{\left(c_{i}+1\right)}^{2}-4c_{i}x}}\right]}^{1/(\alpha -1)}, \end{array}$$

where $c_{i}=\frac{{\left(2\alpha -3\right)}^{2}}{\left(\alpha -1\right)\left(\alpha -3\right)}M\left(1-2\tau_{i}^{1-\alpha }\right)$ (Jo et al., 2019).

A total of 1,000 sequences with a power-law inter-event time distribution were generated with different parameters according to Equation (30). The power-law exponent α, memory coefficient *M*, and the number of events for each event sequence are randomly and independently drawn from 2.1 ≤ α ≤ 2.9, −1/3 ≤ *M* ≤ 1/3, and 50 ≤ *n* ≤ 500, respectively. The initial inter-event time was randomly drawn from 1 to 2. Depending on the power-law exponent and memory coefficient, the burstiness ranged from 0.297 to 0.962.

**Figure 4:**
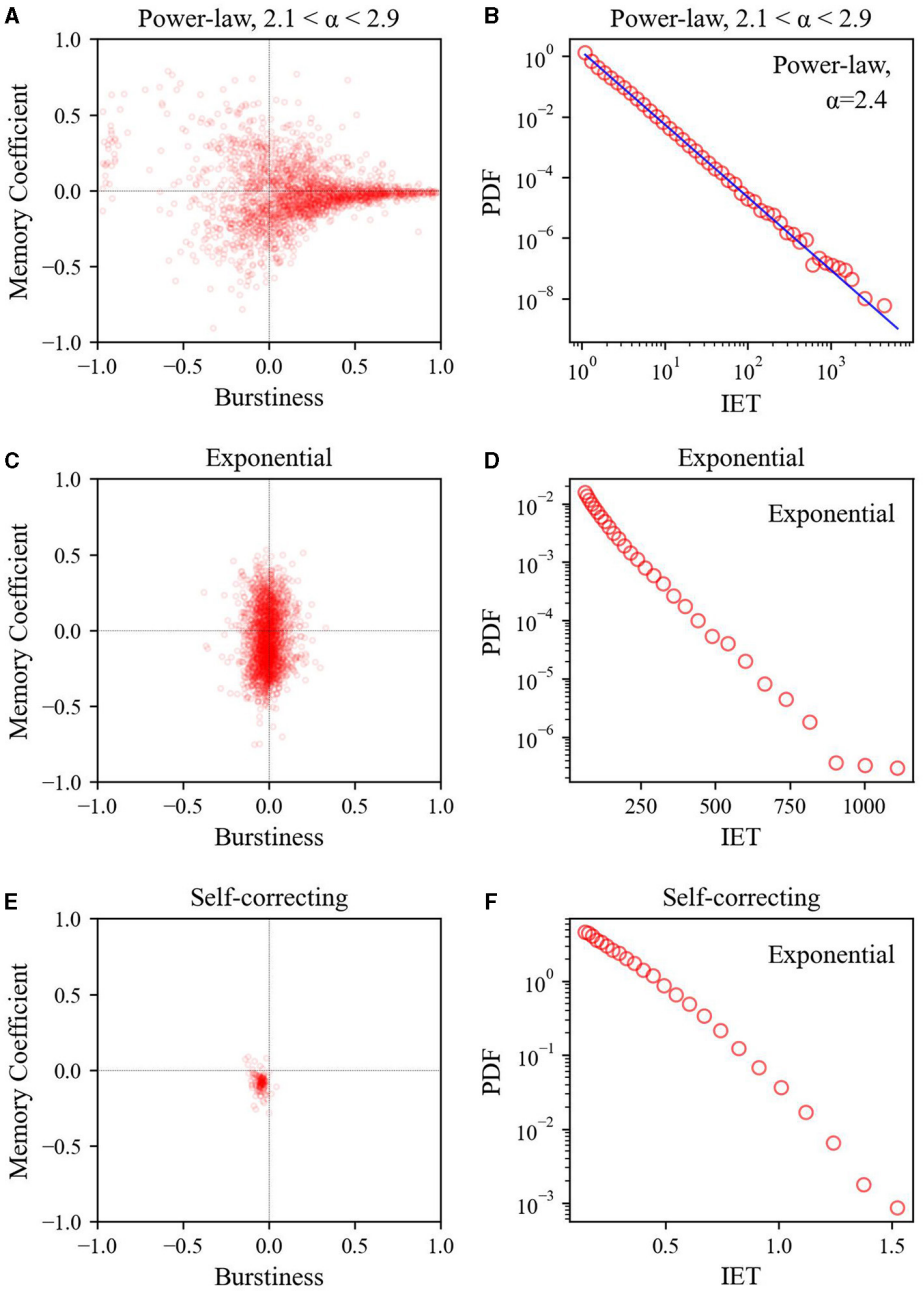
Relationship between burstiness and memory coefficient (left) and inter-event time distribution (right) across three synthetic datasets: **(A, B)** power-law inter-event time, **(C, D)** exponential inter-event time, and **(E, F)** self-correcting process. For calculating the inter-event time distribution, logarithmic binning was employed.

As depicted in Figure 4, the power-law inter-event time datasets exhibit pronounced dispersion toward the region of larger burstiness and memory coefficients (*B* and *M* scatter plots). Moreover, these datasets show a power-law inter-event time distribution with exponent values α = 2.4 close to the average within the range of exponents 2.1 < α < 2.9.

#### 4.1.2 Exponential inter-event time distribution

The exponential inter-event time distribution with mean μ is defined as *P*(τ) = μ^−1^*e*^−τ/μ^ and the corresponding cumulative distribution function is *F*(τ) = 1 − *e*^−τ/μ^ and the relationship between the parameter and memory coefficient is *r* = 4*M*. After substituting the inter-event time distribution into Equation (13), we obtain the next inter-event time τ_*i*+1_ from a given previous inter-event time τ_*i*_ and random number *x* in 0 ≤ *x* < 1 as follows:


(31) $$\begin{array}{l} \tau_{i+1}=\mu \ln\left[\frac{2c_{i}}{c_{i}+1-\sqrt{{\left(c_{i}+1\right)}^{2}-4c_{i}x}}\right], \end{array}$$

where $c_{i}=4M\left(1-2e^{-\tau_{i}/\mu }\right)$ (Jo et al., 2019).

A total of 1,000 sequences with an exponential inter-event time distribution were generated using different parameters, according to Equation (31). The mean inter-event time μ, memory coefficient *M*, and the number of events *n* for each event sequence were randomly and independently drawn from 1 ≤ μ ≤ 100, −1/3 ≤ *M* ≤ 1/3, and 50 ≤ *n* ≤ 500, respectively. The initial inter-event time was set to μ for each event sequence.

As illustrated in Figure 4, the *B* and *M* scatter plots of the exponential inter-event time datasets show that *B* values are concentrated in the lower range, whereas *M* values exhibit a broader distribution spread both above and below. This contrasts with the self-correcting process datasets, where the *B* and *M* scatter plots show that both *B* and *M* clustered at ~0. Although both datasets have an exponential inter-event time distribution, their heterogeneity differs owing to variations in the relationship between *B* and *M*. Even with an exponential inter-event time distribution, appropriately shuffling inter-event times can generate event sequences with temporal heterogeneity (i.e., burst) characteristics. We examine this difference further later, as it plays a role in generating variations in performance.

### 4.2 Real-world datasets

We adopted four real-world datasets to evaluate the models: the *Retweets, StackOverflow, Financial Transaction*, and *911 Calls* datasets. The Retweets dataset (Zhao et al., 2015) contains sequences of tweets and follow-up tweets. The original datasets contained three categories, based on the number of followers. The StackOverflow dataset (Leskovec and Krevl, 2014) contains each user's reward history, that is, the timestamp of users receiving the badge and the type of the badge. The Financial Transaction dataset (Du et al., 2016) includes raw order book records from the New York Stock Exchange (NYSE) for a stock in one day, with a millisecond-level time granularity. The events correspond to two types of actions: buy and sell orders. The 911 Calls datasets^1^ contains emergency phone call records for Montgomery County, PA. This dataset contains information such as calling times and location, and we conducted aggregation based on location, utilizing zip codes as identifiers. The dataset covers a five-year period, which is a relatively extensive time frame for prediction purposes. Therefore, we partitioned the data into monthly intervals. Additionally, to ensure statistical significance, we included only those locations where the number of events exceeded 50 in the data.

Although there are other commonly used datasets, the burst and memory-aware characteristics assumed by the BMT model are applicable when the sequence length is sufficiently long. Furthermore, we sampled event sequences in quantities comparable to synthetic data while concurrently excluding sequences with short lengths. The time units for each dataset are as follows: Retweet and StackOverflow datasets are in days, Financial Transaction datasets are in milliseconds, and 911 Calls datasets are in minutes. The statistics of the datasets are displayed in Table 1.

**Figure 5:**
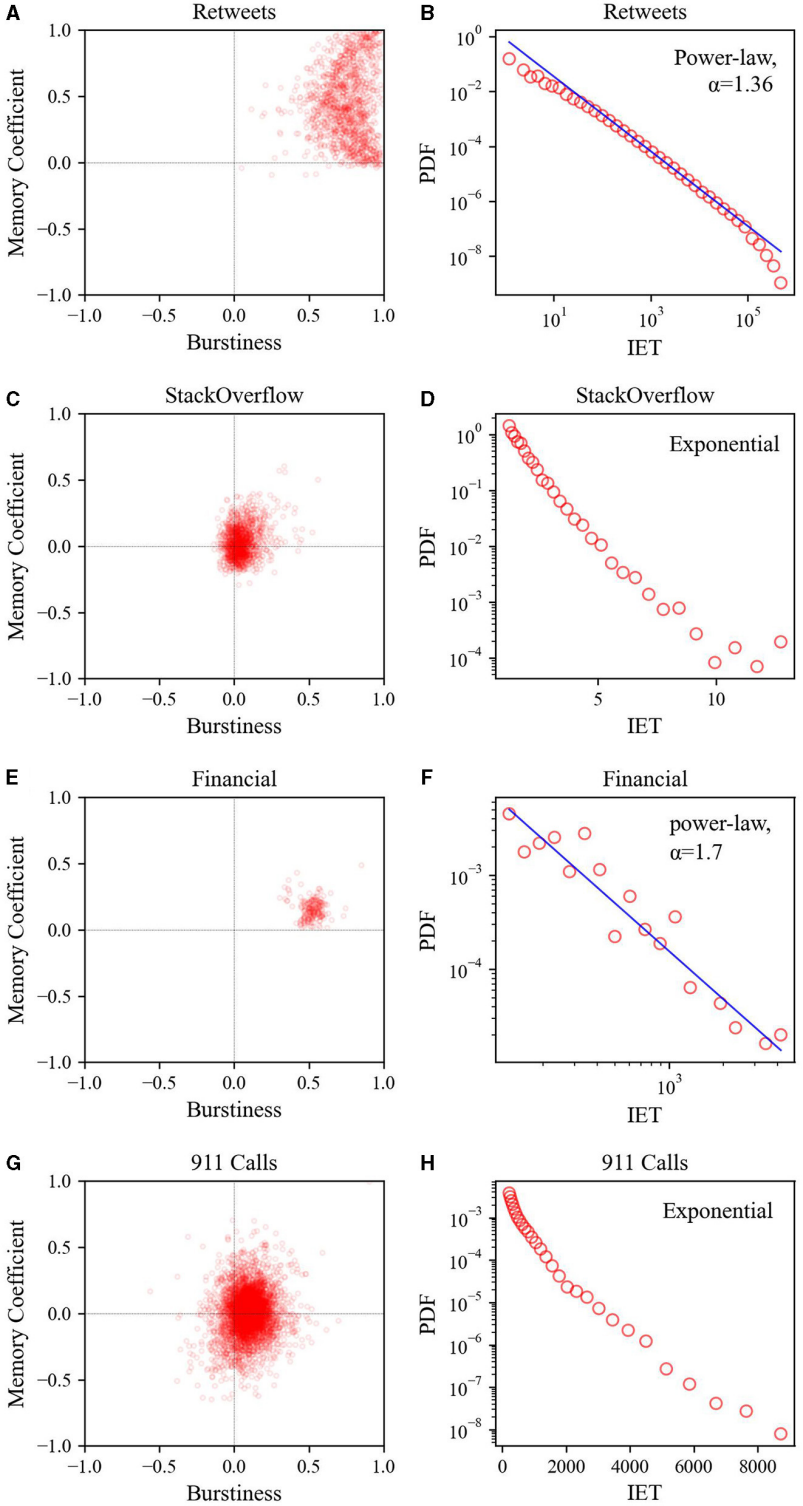
Relationship between burstiness and memory coefficient (left) and inter-event time distribution (right) for four real-world datasets: **(A, B)** Retweets, **(C, D)** StackOverflow, **(E, F)** Financial Transaction, and **(G, H)** 911 Calls. For calculating the inter-event time distribution, logarithmic binning was employed.

As shown in Figure 5, when comparing the Retweets datasets (or Financial Transaction datasets) to the StackOverflow datasets (or 911 Calls datasets), it is evident that the Retweets datasets and Financial Transaction datasets are more temporally heterogeneous. In the *B* and *M* scatter plots, the Retweets datasets (or Financial Transaction datasets) are concentrated in regions with larger values for both *B* and *M*, whereas the StackOverflow datasets (or 911 Calls datasets) are centered around values near 0 for both *B* and *M*. However, when compared to the self-correcting process datasets, the StackOverflow (or 911 Calls datasets) datasets exhibit greater dispersion. Additionally, the inter-event time distribution reveals that the Retweets datasets and Financial Transaction datasets follow a power-law distribution (exponent of 1.36 and 1.70, respectively), whereas the StackOverflow datasets and 911 Calls datasets follow an exponential distribution.

### 4.3 Impact of *B* and *M* embedding and losses

While altering the combination of loss functions during the experimental process, there were five control groups.

1. **BMT-NoE&NoL** (BMT without *B* & *M* embedding and without corresponding losses). The simplest scenario occurs when α_2_ = α_3_ = α_4_ = 0, utilizing only time and event losses. In this case, only event time and intensity were considered.
2. **BMT-NoE&L** (BMT without embedding for *B* & *M*, but with losses for either *B* or *M*). To incorporate the effects of the *B* & *M* losses, we also consider the case of α_2_ = 0, α_3_ > 0, and α_4_ > 0 with time and event losses. Note that the case for α_2_ > 0 relates to predicting the discretized on-hot *B* & *M*, and hence it is not applicable in this scenario.
3. **BMT-E&NoL** (BMT without losses related to *B* & *M*, but with embedding for *B* & *M*). The control group examines the impact of loss for *B* & *M*; the representation vector remains consistent with the BMT model, as shown in Equation (18), but without $L_{B,M}$, $L_{B}$, and $L_{M}$, that is, α_2_ = α_3_ = α_4_ = 0.
4. **BMT-B** (BMT with *B* embedding only and the corresponding loss). In the case where only *B* is embedded and the model is trained, the loss is also computed exclusively based on *B* as α_2_ = 0, α_3_ > 0, and α_4_ = 0 with time and event losses.
5. **BMT-M** (BMT with *M* embedding only and corresponding loss). In the case where only *M* is embedded and the model is trained, the loss is also computed exclusively based on *M* as α_2_ = α_3_ = 0, and α_4_ > 0 with time and event losses.

## 5 Results and discussion

We tested several hyperparameters for both the BMT and THP models and chose the configuration that yielded the best validation performance. The hyperparameters are as follows: the number of bins for discretization (*b*) is set to 40, mini-batch size is 16, dropout rate is 0.1, embedding dimensions (*d* and *d*_*H*_) are both 128, self-attention dimensions (*d*_*K*_ and *d*_*V*_) are 32, with eight layers in the encoder and 8 heads. For the loss function, hyperparameters were fine-tuned, mainly as follows: α_1_ = 1*e*3, α_2_ = 4*e*3, α_3_ = α_4_ = 1*e*4. We employed the ADAM (adaptive moment estimation) optimizer with hyperparameters β set to (0.9, 0.999). Regarding the learning rate, we utilized PyTorch StepLR, initializing it at 1e-4 and reducing the learning rate by a factor of 0.9 every 15 steps.

The performance evaluation results for different models across diverse datasets are presented in Table 2. The results indicate that BMT achieves superior performance compared to THP and other control models in terms of the root mean squared error (RMSE) of the event times and log-likelihood. The main metric, RMSE, is a unit-adjusted value obtained by taking the square root of Equation (21). It measures how much predicted event times of the model differ from the actual event times. However, RMSE has a drawback, especially in the case of heterogeneous data, where it can perform well by accurately predicting large values while potentially struggling with smaller ones. To address this limitation, we introduce the event log-likelihood, defined in Equation (22), as a second metric. This metric arises when probabilistically modeling event sequences using the intensity function λ derived from Equation (1). A higher likelihood of the intensity function calculated with predicted event times of the model indicates that the model better probabilistically mimics the actual event sequence. Consequently, larger values of this metric correspond to better performance. Additionally, when considering the *B* and *M* losses in Equation (26), they represent how well the model captures discretized burstiness and discretized memory coefficients. Smaller values of these losses indicate better performance in replicating these aspects.

**Table 2:** Performance evaluation results across diverse datasets for different models.

**Dataset**	**Model**	**RMSE of time**	**LL**	**CE of BM**
Power- law	THP	238.0	−2.303	N/A
	BMT-NoE&NoL	66.6	−2.451	N/A
	BMT-NoE&L	68.2	−2.493	N/A
	BMT-E&NoL	82.0	−2.348	12.46
	BMT-B	107.6	−2.712	N/A
	BMT-M	52.3	−2.730	N/A
	BMT	**40.5**	**−2.302**	**8.05**
Exponential	THP	1,973.7	−19.910	N/A
	BMT-NoE&NoL	158.7	−6.486	N/A
	BMT-NoE&L	208.8	−15.640	N/A
	BMT-E&NoL	102.2	−5.633	12.70
	BMT-B	106.1	−7.201	N/A
	BMT-M	140.8	−9.912	N/A
	BMT	**80.1**	**−5.171**	**5.77**
Self-correcting	THP	0.184	0.200	N/A
	BMT-NoE&NoL	0.192	−0.281	N/A
	BMT-NoE&L	0.185	0.329	N/A
	BMT-E&NoL	0.183	0.592	7.30
	BMT-B	0.198	−0.425	N/A
	BMT-M	0.209	−0.456	N/A
	BMT	**0.181**	**0.605**	**5.43**
Retweets	THP	36,080.8	−9.01	N/A
	BMT-NoE&NoL	16,360.8	−111.13	N/A
	BMT-NoE&L	16,362.9	−110.95	N/A
	BMT-E&NoL	16,257.7	**−8.14**	28.92
	BMT-B	16,090.8	−16.21	N/A
	BMT-M	16,266.5	−11.19	N/A
	BMT	**15,825.8**	−11.28	**2.70**
Stack Overflow	THP	127.0	−0.373	N/A
	BMT-NoE&NoL	0.658	**−0.266**	N/A
	BMT-NoE&L	**0.643**	−0.277	N/A
	BMT-E&NoL	0.726	−0.339	17.81
	BMT-B	0.858	−0.718	N/A
	BMT-M	3.969	−0.505	N/A
	BMT	0.663	−0.358	**6.38**
Financial	THP	38.13	−1.826	N/A
	BMT-NoE&NoL	44.26	−11.843	N/A
	BMT-NoE&L	62.72	−11.759	N/A
	BMT-E&NoL	38.39	−2.104	7.39
	BMT-B	37.93	−1.848	N/A
	BMT-M	77.58	−1.796	N/A
	BMT	**37.92**	**−1.775**	**4.41**
911 Calls	THP	6,183.4	−7.190	N/A
	BMT-NoE&NoL	358.3	−17.662	N/A
	BMT-NoE&L	469.3	−41.818	N/A
	BMT-E&NoL	342.4	**−6.608**	28.49
	BMT-B	353.4	−6.832	N/A
	BMT-M	364.8	−6.835	N/A
	BMT	**339.6**	−6.883	**8.56**

RMSE, root mean squared error; LL, log-likelihood; CE, cross entropy; B, burstiness; M, memory coefficient. The bold value indicates the metric of the model with the best performance for each individual dataset.

In particular, as the data became more heterogeneous, performance improvement became more pronounced. In synthetic datasets, the performance enhancement of the BMT model was greater for power-law inter-event time data than for self-correcting data, which is a less heterogeneous exponential inter-event time distribution (see Figure 4). Similarly, in real-world datasets, the overall performance of the BMT model was superior in the Retweets dataset, which exhibited a more power-law inter-event time distribution, compared to the StackOverflow datasets with a less heterogeneous exponential inter-event time distribution (see Figure 5).

When compared to the BMT-NoE&L model with respect to the RMSE of the event times, the BMT model shows that superior performance across all datasets except StackOverflow. This suggests that the inclusion of *B* & *M* embedding processes aids in augmenting the performance of the model by enabling the encoder to grasp the burst structure of event sequences. Compared with the BMT-E&NoL model, the BMT model demonstrates enhanced performance across all datasets, indicating that the integration of *B* & *M* losses into the overall loss function contributes to the improved performance of the model. Even in the prediction of one-hot discretized *B* & *M*, it can be observed that including *B* & *M* losses contributes to a reduction in cross entropy. No significant differences in performance were observed between the BMT-NoE&NoL and BMT-NoE&L models. This suggests that the incorporation of *B* & *M* losses is less significant in the absence of *B* & *M* embedding.

**Figure 6:**
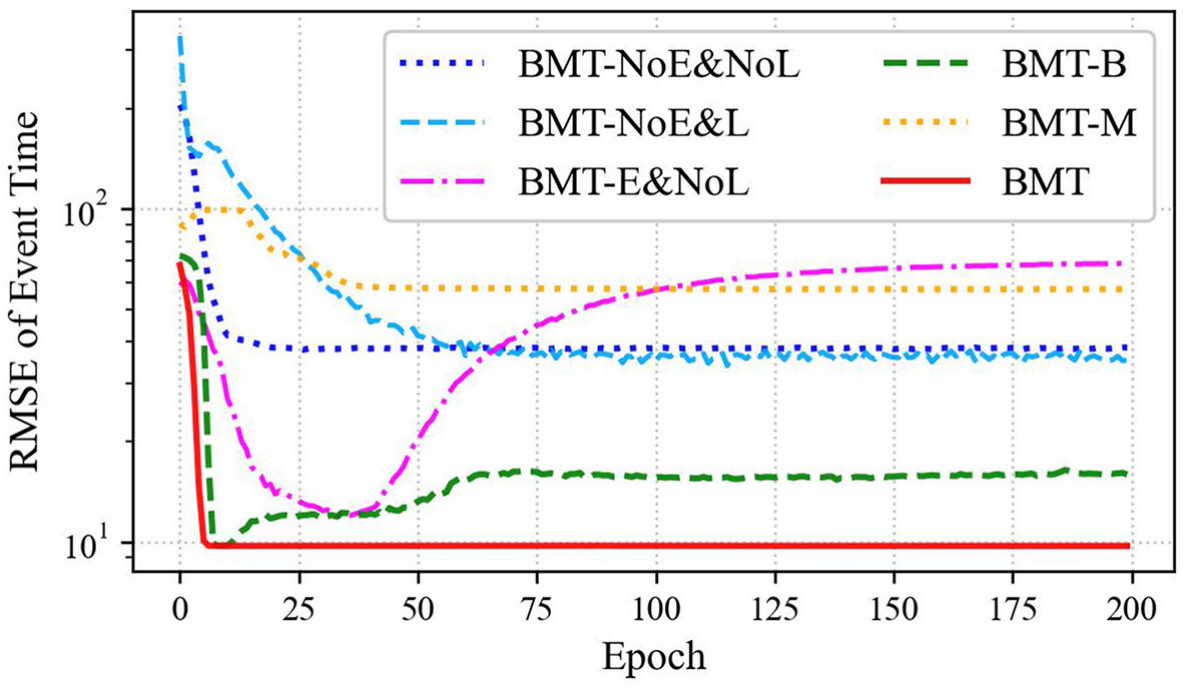
Training curves of RMSE for event times fitted on Financial Transaction datasets are presented for various BMT model scenarios: BMT-NoE&NoL, BMT-NoE&L, BMT-E&NoL, BMT-B, BMT-M, and the standard BMT model.

Summarizing the aforementioned findings, it is evident that both *B* & *M* embedding and *B* & *M* losses contribute to performance enhancement. Excluding either of these components would likely impede the attainment of a substantial performance improvement, comparable to that observed with the BMT model. If either of the *B* embedding or *M* embedding is omitted, a significant performance improvement comparable to that of the BMT model cannot be expected. This was substantiated by comparing the BMT model with the BMT-B and BMT-M models, which revealed the superior performance of the BMT model across all datasets. These results can also be observed in the training curves shown in Figure 6.

We also conducted experiments on mixed synthetic datasets, the results of which are presented in Table 3. The mixed synthetic datasets comprised a combination of three individual datasets: power-law, exponential, and self-correcting datasets. However, when separately examining the RMSE of event time and log-likelihood, the performance of the original BMT model appeared slightly inferior compared to some of the control BMT models, demonstrating an overall superior performance when considering both metrics together.

**Table 3:** Performance evaluation results for the mixed synthetic datasets: power-law, exponential, and self-correcting datasets.

**Model**	**RMSE of time**	**LL**	**CE of BM**
THP	1,338.65	−14.190	N/A
BMT-NoE&NoL	261.20	−9.015	N/A
BMT-NoE&L	**64.19**	−8.228	N/A
BMT-E&NoL	77.14	**−4.414**	12.593
BMT-B	64.74	−7.439	N/A
BMT-M	71.31	−10.476	N/A
BMT	66.98	−4.748	**6.269**

The bold value indicates the metric of the model with the best performance for each individual dataset.

In summary, the BMT model demonstrates improved performance on heterogeneous data owing to its capability to capture heterogeneous characteristics through the embedding of *B* & *M*, combined with the inclusion of corresponding loss functions.

The BMT model has two limitations. First, in cases where the event sequence length is short, the incorporation of *B* and *M* into the BMT model may result in reduced effectiveness. This aspect originates from the statistical characteristics of *B* and *M*, because their meaningful representation is hindered by fluctuations and noise, particularly when the number of events is small. In the BMT model, during the calculation of sliding *B* and *M* values, masking was applied to exclude the first three events. However, considering that temporal heterogeneity becomes a meaningful characteristic only when the length of the event sequence is sufficiently long, this limitation can be viewed as unavoidable.

The second limitation is the inability to consider event types, which will be addressed in future studies. To account for event types, it is necessary to reflect the correlation structure between inter-event times and event types to generate synthetic data and subsequently test the model using these data. In the context of performance enhancement, the improvement of the BMT model over the THP model can also be attributed to the fact that the BMT model does not embed event types. This allows the model to focus more on predicting the event times. Because the BMT-NoE&NoL model is analogous to a version of the THP model that does not consider event types, comparing the performance of the BMT-NoE&NoL model with the BMT model would provide a more equitable assessment. However, upon comparing the BMT-NoE&NoL model with the BMT model, it becomes evident that the BMT model exhibits superior performance across all datasets, except for StackOverflow.

## 6 Conclusion

Our study addresses the challenges presented by bursty temporal patterns in event sequences across various domains. By leveraging recent advancements in predicting event sequences using Transformer models based on the Hawkes process with self-attention mechanisms, we introduced a Burst and Memory-aware Transformer (BMT) model. This model effectively captures the nuances of burst patterns by embedding burstiness and memory coefficient within its self-attention module. The incorporation of a specialized loss function tailored for burstiness and memory coefficient further refines the model's predictive capabilities.

Through comprehensive numerical experiments conducted on a diverse array of synthetic and real-world datasets encompassing various scenarios, we validated the outstanding performance of the BMT model by comparing it with the existing models and control groups. This is particularly evident in scenarios involving heterogeneous data, such as power-law inter-event time distributions. Hence, the explicit consideration of burst-related parameters within the Transformer contributes to a deeper comprehension of complex event sequences, ultimately leading to an enhanced predictive performance. In future work, we will focus on integrating a multitude of insights from complex systems into the development of deep neural network models for temporal data.

## Data availability statement

The datasets presented in this study can be found in online repositories. The names of the repository/repositories and accession number(s) can be found in the article/supplementary material. The synthetic data can be found here: https://github.com/bh0903lee/BMT-synthetic-datasets.

## Author contributions

BL: Conceptualization, Data curation, Formal analysis, Investigation, Methodology, Resources, Software, Validation, Visualization, Writing—original draft, Writing—review & editing. J-HL: Formal analysis, Resources, Software, Writing—review & editing. SL: Formal analysis, Resources, Software, Writing—review & editing. CK: Formal analysis, Resources, Software, Writing—review & editing.

## Footnotes

^1^The dataset is available on https://www.kaggle.com/datasets/mchirico/montcoalert/.
